# Genome-Wide Identification and Characterization of the CBL Gene Family in *Ziziphus jujuba* var. *spinosa*

**DOI:** 10.3390/genes17091121

**Published:** 2026-09-15

**Authors:** Yilong Lu, Yong Fan, Kun Liu, Jian Wen, Jialin Sun, Wensong Sun

**Affiliations:** Liaoning Research Institute of Cash Crops, Liaoning Academy of Agricultural Sciences, Liaoyang 111000, China; yilonglu2021@163.com (Y.L.); fanyongzh@126.com (Y.F.); yuanteng1986@126.com (K.L.); zhongyaocaiwenjian@163.com (J.W.); joyce8813@126.com (J.S.)

**Keywords:** CBL, *Ziziphus jujuba* var. *spinosa*, saline–alkali stress, expression analysis, protein–protein interaction

## Abstract

Background: Calcineurin B-like (CBL) protein-mediated calcium signaling represents a core regulatory pathway underlying plant abiotic stress adaptation. *Ziziphus jujuba* var. *spinosa* (sour jujube) is a perennial woody species with remarkable saline–alkali and drought tolerance, yet the *CBL* gene family in this species has not been systematically characterized. Methods: We performed genome-wide identification and characterization of the *CBL* gene family in sour jujube through integrated bioinformatic analyses, transcriptomic profiling, qRT-PCR, and yeast two-hybrid (Y2H) assays, with the annotation of one gene manually corrected based on molecular cloning and transcriptome validation. Results: Ten non-redundant *ZjCBL* genes were identified and grouped into three phylogenetic clades. Synteny and selection pressure analyses identified two collinear gene pairs that have undergone strong purifying selection. All ZjCBL proteins contain canonical EF-hand motifs and are predicted to be mainly localized to the plasma membrane, with ZjCBL4 and ZjCBL9 harboring additional N-terminal transmembrane helices. The promoter regions of *ZjCBL* genes are enriched in hormone- and stress-responsive cis-acting elements. *ZjCBL1*, *ZjCBL2*, *ZjCBL3*, and *ZjCBL5* were highly expressed across multiple tissues, and *ZjCBL1* exhibited sustained upregulation under long-term saline–alkali stress, concurrent with the accumulation of osmoprotectants. Y2H assays confirmed that ZjCBL1 interacts with two ZjCIPK proteins, exhibiting a stronger interaction signal with ZjCIPK13. Conclusions: This study provides the first systematic characterization of the *CBL* gene family in sour jujube and revises the annotation of *ZjCBL1*, establishing *ZjCBL1* as an important component of the ZjCBL–ZjCIPK signaling network and offering candidate gene resources for molecular breeding to enhance stress tolerance in fruit crops.

## 1. Introduction

Abiotic stresses represent major constraints to plant growth and crop productivity worldwide, particularly for perennial fruit crops grown in marginal lands [1]. As a ubiquitous secondary messenger in plant cells, calcium ions (Ca^2+^) serve as a core regulator of growth, development, hormone signal transduction, and adaptive responses to diverse abiotic stresses, such as drought, salinity, extreme temperatures, and heavy-metal toxicity [2,3]. Transient fluctuations in cytosolic Ca^2+^ concentration are sensed and decoded by a suite of Ca^2+^ sensor proteins. In higher plants, four major families of Ca^2+^ sensors have been characterized: calmodulins (CaMs), calmodulin-like proteins (CMLs), calcium-dependent protein kinases (CDPKs), and calcineurin B-like proteins (CBLs) [4,5]. As a plant-specific Ca^2+^ sensor family, CBL proteins exert their regulatory functions by associating with CBL-interacting protein kinases (CIPKs) to form functional signaling modules. These CBL-CIPK modules modulate ion homeostasis, osmotic adjustment, reactive oxygen species (ROS) scavenging, and stress-responsive gene expression, constituting a core signaling cascade underlying plant abiotic stress adaptation [6,7].

CBL proteins exhibit high structural conservation across land plants. Most family members harbor 3–4 canonical EF-hand calcium-binding motifs responsible for specific Ca^2+^ recognition and coordination [8]. Most CBL proteins carry conserved N-terminal myristoylation and palmitoylation sites that play an important role in determining their subcellular localization and mediate the anchoring of CBL-CIPK complexes to the plasma membrane or tonoplast [9,10]. A conserved PFPF motif located at the C-terminus is essential for the specific physical interaction between CBLs and CIPKs [10]. Unlike CDPKs, CBL proteins do not exhibit intrinsic kinase catalytic activity. Upon Ca^2+^ binding, CBLs associate with CIPKs and relieve autoinhibition of the CIPK kinase domain, thereby activating CIPK serine/threonine kinase activity. The activated CBL-CIPK complexes subsequently transduce Ca^2+^ signals via the phosphorylation of downstream target proteins [11].

Functional characterization across species has demonstrated that CBL-CIPK modules participate broadly in plant responses to diverse abiotic stresses, including salt, drought, cold, low-potassium, and heavy metal stress [12]. The best-characterized example is the salt overly sensitive (SOS) pathway in *Arabidopsis thaliana* (*A. thaliana*): the *AtCBL4*-*AtCIPK24* complex activates the plasma membrane Na^+^/H^+^ antiporter SOS1 to extrude excess cytosolic Na^+^, thereby maintaining ion homeostasis and conferring salt tolerance [13,14]. In *Triticum turgidum*, TtCBL2 is rapidly upregulated under drought stress and mediates drought signaling via an abscisic acid (ABA) signaling pathway [15]. Overexpression of *OsCBL1* in *Oryza sativa* (*O. sativa*) enhances cold tolerance by promoting DNA damage repair, modulating ROS homeostasis, and increasing cuticular wax deposition [16]. Additionally, plasma membrane-localized AtCBL1/9-CIPK and tonoplast-localized AtCBL2/3-CIPK cascades regulate K^+^ uptake and vacuolar compartmentalization via activation of distinct K^+^ channels [17]. Heterologous expression of sugarcane ScCBL genes in *Escherichia* coli has also been shown to enhance Cu^2+^ tolerance [18]. To date, genome-wide characterization of the *CBL* gene family has been reported in a wide range of plant species, including *Citrus sinensis* (8 members), *Carya illinoinensis* (9), *Camellia sinensis* (8), *Magnolia biondii* (6), *Mesembryanthemum crystallinum* (9), *Beta vulgaris* (7), *Cucumis sativus* (7), *Lonicera japonica* (6), *Zea mays* (12), *Medicago sativa* (23), and *Chenopodium quinoa* (16) [19,20,21,22,23,24,25,26,27,28,29]. Collectively, these findings support the critical roles of CBL proteins in stress tolerance and highlight the extensive functional divergence of the family across plant lineages.

*Ziziphus jujuba* var. *spinosa* (sour jujube) is a perennial shrub in the Rhamnaceae family, widely distributed across arid, semi-arid, and saline–alkali regions of northern China. This species has high ecological, economic, and medicinal value. Its dried mature seeds, commercially known as Semen Ziziphi Spinosae, are abundant in saponins, flavonoids, alkaloids, and other bioactive compounds, and represent a classic traditional Chinese medicine widely used for insomnia management. Benefiting from its robust root system, sour jujube exhibits strong tolerance to drought, salinity, cold, and nutrient-poor soils. It is therefore widely deployed for soil and water conservation, and also serves as a valuable stress-resistant germplasm resource for jujube molecular breeding [30,31]. The recent release of the sour jujube reference genome has enabled genome-wide systematic analysis of gene families in this species [32]. However, despite the established importance of CBL-mediated calcium signaling in plant stress adaptation, the *CBL* family remains uncharacterized in sour jujube. The full repertoire of *ZjCBL* members, their phylogenetic relationships, expression profiles across tissues and developmental stages, and transcriptional responses to saline–alkali and other abiotic stresses have yet to be systematically examined. Moreover, the downstream CIPK partners of ZjCBL proteins are entirely unknown. These gaps limit our understanding of calcium-mediated stress tolerance in woody plants and the utilization of this germplasm for molecular breeding of stress-tolerant jujube.

In the present study, we performed genome-wide identification and comprehensive characterization of the *CBL* gene family in sour jujube using a combination of bioinformatic analysis and molecular cloning, with manual curation of genome annotation errors. We systematically analyzed the fundamental features of *ZjCBL* genes, including gene number, physicochemical properties, chromosomal localization, gene structure, conserved motifs, cis-acting regulatory elements, and phylogenetic relationships. We further profiled their expression patterns across diverse tissues, fruit developmental stages, and salt, drought, and combined saline–alkali stress treatments to identify key stress-tolerance candidate genes. The physical interaction between ZjCBL1 and two candidate ZjCIPKs was subsequently validated via Y2H assay. Our findings lay a foundational framework for dissecting the stress tolerance mechanism of sour jujube and provide candidate gene resources for molecular breeding of stress-tolerant jujube and other fruit tree species.

## 2. Materials and Methods

### 2.1. Plant Materials and Treatments

All plant materials used in this study were sour jujube seedlings grown in a climate chamber under controlled conditions (16 h light/8 h dark, 28 °C, 60% relative humidity). To examine differential gene expression under saline–alkali stress, newly germinated seedlings were treated with a combined saline–alkali solution (150 mM Na^+^, pH 9.8); control seedlings were maintained in pure water (pH 6.8). Following 15 d of continuous stress, root and leaf tissues were collected for differential expression analysis. In a separate experiment, healthy 25-day-old seedlings were exposed to a higher concentration of saline–alkali solution (200 mM Na^+^, pH 10.3) to monitor dynamic changes in transcript levels and physiological/biochemical parameters. Tissue samples were harvested at 2, 4, 6, 8, and 10 days after stress initiation. All saline–alkali solutions were prepared by mixing NaCl, Na_2_SO_4_, NaHCO_3_, and Na_2_CO_3_. The detailed composition of each solution, including the concentrations of individual salts, is provided in Table S1. The collected samples were immediately frozen in liquid nitrogen and stored at −80 °C until further processing.

### 2.2. Genome-Wide Identification and Physicochemical Characterization of ZjCBL Genes

Genome-wide identification of the *ZjCBL* gene family in sour jujube was performed based on the published sour jujube genome assembly [32]. Briefly, whole-genome sequences, predicted protein sequences, coding sequences (CDSs), and genome annotation GFF files of sour jujube were downloaded from the NCBI database (BioProject: PRJNA974227) and the Figshare platform (https://figshare.com/s/ad5d747ccc2ccbb2b65b, accessed on 2 November 2025). Experimentally validated *AtCBL* and *OsCBL* protein sequences were used as queries for BLASTP via TBtools (v2.056) searches against the sour jujube protein dataset to screen candidate ZjCBL proteins. Following initial screening, the presence of conserved EF-hand calcium-binding domains in all candidate proteins was confirmed using the NCBI Conserved Domain Database (CDD) server (https://www.ncbi.nlm.nih.gov/Structure/cdd/wrpsb.cgi, accessed on 15 November 2025) and the SMART tool (http://smart.embl-heidelberg.de/, accessed on 15 November 2025). Ultimately, all non-redundant candidate sequences were validated by cross-referencing with our in-house transcriptome data and Sanger sequencing results of molecular cloning. Physicochemical properties of the confirmed ZjCBL proteins were predicted using the ExPASy ProtParam tool (https://www.expasy.org/, accessed on 22 December 2025). In parallel, transmembrane helices were predicted using the TMHMM v2.0 server (https://services.healthtech.dtu.dk/services/TMHMM-2.0/, accessed on 23 December 2025), and subcellular localization was inferred via the Plant-mPLoc v2.0 module of the Cell-PLoc 2.0 package (http://www.csbio.sjtu.edu.cn/bioinf/plant-multi/, accessed on 23 December 2025) using default parameters.

### 2.3. Phylogenetic, Collinearity, and Selection Pressure Analyses of ZjCBL Genes

Multiple sequence alignment of the 10 identified ZjCBL proteins, 10 *A. thaliana* CBL proteins, and 10 *O. sativa* CBL proteins was conducted using MEGA 7.0 (Table S2). The best-fit amino acid substitution model was determined using the “Find Best DNA/Protein Models” function in MEGA 7.0, with the optimal model selected based on the Bayesian Information Criterion (BIC). Maximum likelihood (ML) phylogenetic trees were constructed with 1000 bootstrap replicates, and all other parameters were set to default values. Chromosomal localization and synteny analysis of *ZjCBL* genes were performed in TBtools (v2.056) based on the genome assembly and corresponding annotation files. Tandem and segmental gene duplication events were identified using MCScanX (https://github.com/wyp1125/MCScanX, accessed on 8 January 2026), and the non-synonymous (Ka) and synonymous (Ks) substitution rates of duplicated gene pairs were calculated. Evolutionary selection pressure on duplicated *ZjCBL* genes was inferred based on Ka/Ks ratios.

### 2.4. Analyses of Conserved Motifs, Gene Structure, and Promoter Cis-Acting Elements

Conserved protein motifs of ZjCBL proteins were predicted using the MEME online tool (v5.5.9, https://meme-suite.org/meme/tools/meme, accessed on 8 January 2026), with the maximum number of motifs set to 5 and motif length restricted to 6–50 amino acids. An integrated plot combining the phylogenetic tree, conserved motif distribution, domain architecture, and exon–intron structure was generated using TBtools. The 2000 bp genomic sequences upstream of the transcription start site (TSS) of each *ZjCBL* gene were extracted in TBtools, and cis-acting regulatory elements within these promoter regions were annotated using the PlantCARE database (http://bioinformatics.psb.ugent.be/webtools/plantcare/html/, accessed on 13 January 2026).

### 2.5. Functional Annotation and miRNA Target Prediction

Gene Ontology (GO) functional annotation of all ZjCBL protein sequences was conducted using the EggNOG-mapper v2 tool (http://eggnog-mapper.embl.de/, accessed on 21 January 2026). KEGG Orthology (KO) identifiers were assigned via the BlastKOALA server (https://www.kegg.jp/blastkoala/, accessed on 20 January 2026) to infer potential roles of ZjCBLs in metabolic and signaling pathways. GO and KEGG pathway enrichment analyses for the *ZjCBL* gene set were performed in TBtools, with the entire sour jujube gene set as the statistical background. For miRNA target prediction, published sour jujube miRNA sequences were downloaded from the PmiREN database (https://www.pmiren.com/, accessed on 22 January 2026) to build a local reference library. Putative miRNA targeting sites in *ZjCBL* coding sequences were predicted using the psRNATarget server with default parameters, with full-length CDSs used as target sequences.

### 2.6. Expression Pattern Analysis

For RNA-seq analysis, three independent biological replicates were established for each treatment and time point, with each replicate comprising pooled tissue samples from three morphologically uniform seedlings—the same sample design used for quantitative real-time PCR (qRT-PCR) validation. Following quality filtering and read mapping, FPKM (fragments per kilobase of transcript per million mapped reads) values were calculated for each *ZjCBL* gene to systematically characterize their tissue-specific expression profiles and responses to different abiotic stresses. Specifically, raw reads were filtered using fastp (v0.23.4) to remove adapter and primer sequences, discard reads with average quality score below Q5, and eliminate reads containing more than five N bases. Clean reads were mapped to the sour jujube reference genome [32] using HISAT2 (v2.2.1). Gene expression levels were quantified with StringTie (v2.2.0) and normalized as FPKM values. Pairwise expression correlations among *ZjCBL* genes were calculated based on FPKM values using SPSS (v26.0). Gene expression heatmaps and correlation heatmaps were visualized in TBtools, and boxplots for expression pattern visualization were generated in RStudio (v2023) using the R packages *ggplot2* (v3.4.4), *pheatmap* (v1.0.12), and *ggpubr* (v0.6.0).

For qRT-PCR validation, total RNA was extracted from all harvested samples using a plant total RNA extraction kit. First-strand cDNA was synthesized with the TaKaRa PrimeScript RT reagent Kit (Takara Bio, Kusatsu, Japan) according to the manufacturer’s instructions. Gene-specific primers were designed using Primer Premier 5.0 based on *ZjCBL* coding sequences. qRT-PCR reactions were carried out using SYBR Green PCR Master Mix, with *ZjACTIN7* (*ZspiChr8G00161390*) serving as the endogenous reference gene. Each biological replicate was measured individually, yielding three independent data points per treatment group. Relative expression levels of *ZjCBL* genes were quantified using the 2^−ΔΔCt^ method. Statistical significance between control and treatment groups was assessed using Student’s *t*-test, with a significance threshold of *p* < 0.05. For multiple comparisons, *p*-values were adjusted using the Benjamini–Hochberg (BH) method to control the false discovery rate (FDR), and both raw and adjusted *p*-values were reported. Primer sequences used in this study are provided in Table S5.

### 2.7. Physiological and Biochemical Measurements

For physiological and biochemical analyses, leaf tissues were ground to a fine powder in liquid nitrogen. Proline (Pro) content was determined using a proline assay kit (JC0601-M, Nanjing Jice Biotechnology Co., Ltd., Nanjing, China) according to the manufacturer’s instructions. Hydrogen peroxide (H_2_O_2_) was measured using a hydrogen peroxide assay kit (JC0201-M, Nanjing Jice Biotechnology Co., Ltd., Nanjing, China). Soluble protein content was quantified using the Coomassie Brilliant Blue G-250 method [33]. Soluble sugar content was determined using a plant soluble sugar assay kit (JC0401-M, Nanjing Jice Biotechnology Co., Ltd., Nanjing, China) with the anthrone–sulfuric acid colorimetric method. All spectrophotometric measurements were performed using a UV-2600 spectrophotometer (Shimadzu, Kyoto, Japan) or an Infinite 200 Pro microplate reader (TECAN, Männedorf, Switzerland).

### 2.8. Yeast Two-Hybrid Assay

Full-length coding sequences of *ZjCBL1*, *ZjCIPK13* (*ZspiChr7G00088720.1*), and *ZjCIPK15* (*ZspiChr11G0020315.1*) were amplified with gene-specific primers (Table S5). The *ZjCBL1* CDS was cloned into the pGBKT7 (bait, BD) vector, and *ZjCIPK13* and *ZjCIPK15* CDSs were separately inserted into the pGADT7 (prey, AD) vector via homologous recombination. All bait-prey combinations, alongside control constructs, were transformed into AH109-competent yeast cells using the lithium acetate transformation method based on the Clontech Gal4 yeast two-hybrid system. Five transformation groups were set: two experimental pairs (*pGBKT7-ZjCBL1* + *pGADT7-ZjCIPK13*, *pGBKT7-ZjCBL1* + *pGADT7-ZjCIPK15*), one autoactivation control (*pGBKT7-ZjCBL1* + empty *pGADT7*), one positive control (*pGBKT7-p53* + *pGADT7-LargeT*), and one negative control (*pGBKT7*-*LaminC* + *pGADT7*-*LargeT*). Transformed yeast suspensions were spread onto SD/-Trp/-Leu (SD-TL) solid plates and incubated at 30 °C for 3–7 days. Single colonies growing on SD-TL plates were randomly picked and verified by yeast colony PCR using the Yeast Colony Rapid Detection Kit (RY8001, Nanjing Ruiyuan Biotechnology Co., Nanjing, China) to confirm successful co-transformation. For autoactivation testing, PCR-positive single colonies were resuspended in sterile water and adjusted to an OD_600_ of 0.2. A 10 μL aliquot of each suspension was spotted onto SD-TL, SD/-Trp/-Leu/-His (SD-TLH), SD/-Trp/-Leu/-His/-Ade (SD-TLHA), and SD-TLHA medium supplemented with X-α-gal, followed by incubation at 30 °C for 3 days. For interaction validation, verified positive colonies were diluted to OD_600_ = 0.2 and prepared into three serial 10-fold dilutions (10^0^, 10^−1^, 10^−2^). A 10 μL drop of each dilution was spotted onto SD-TL, SD-TLH, SD-TLH + X-α-gal, SD-TLHA, and SD-TLHA + X-α-gal selective media. Plates were incubated at 30 °C for 6 days, after which colony growth and blue staining signals were documented.

## 3. Results

### 3.1. Identification and Physicochemical Properties of ZjCBL Genes

A total of 10 non-redundant *CBL* genes were identified in the sour jujube genome and designated *ZjCBL1* to *ZjCBL10* based on their chromosomal order. These 10 *ZjCBL* genes were unevenly distributed on four of the twelve sour jujube chromosomes (Table 1). Chromosome 11 harbored the largest number of *ZjCBL* genes (4 members, highest chromosomal density), followed by chromosome 1 with 3 members; chromosomes 3 and 6 contained 1 and 2 *ZjCBL* genes, respectively. The encoded ZjCBL proteins varied in length from 160 to 312 amino acids, with predicted molecular weights ranging from 18.51 to 36.45 kDa. All ZjCBL proteins had theoretical isoelectric points below 5.5, indicating an overall acidic property of the family. Subcellular localization prediction indicated that all ZjCBL proteins localize to the plasma membrane, while six members (ZjCBL1, ZjCBL5–ZjCBL9) show dual localization to the cytoplasm as well. Sequence validation against the reference genome annotation showed that 9 *ZjCBL* genes were 100% consistent with the annotated sequences, whereas *ZjCBL1* shared only 45.87% identity with the original reference annotation, which was manually corrected in this study based on transcriptome and cloning data.

### 3.2. Phylogenetic Analysis and Synteny Relationships

**Figure 1:**
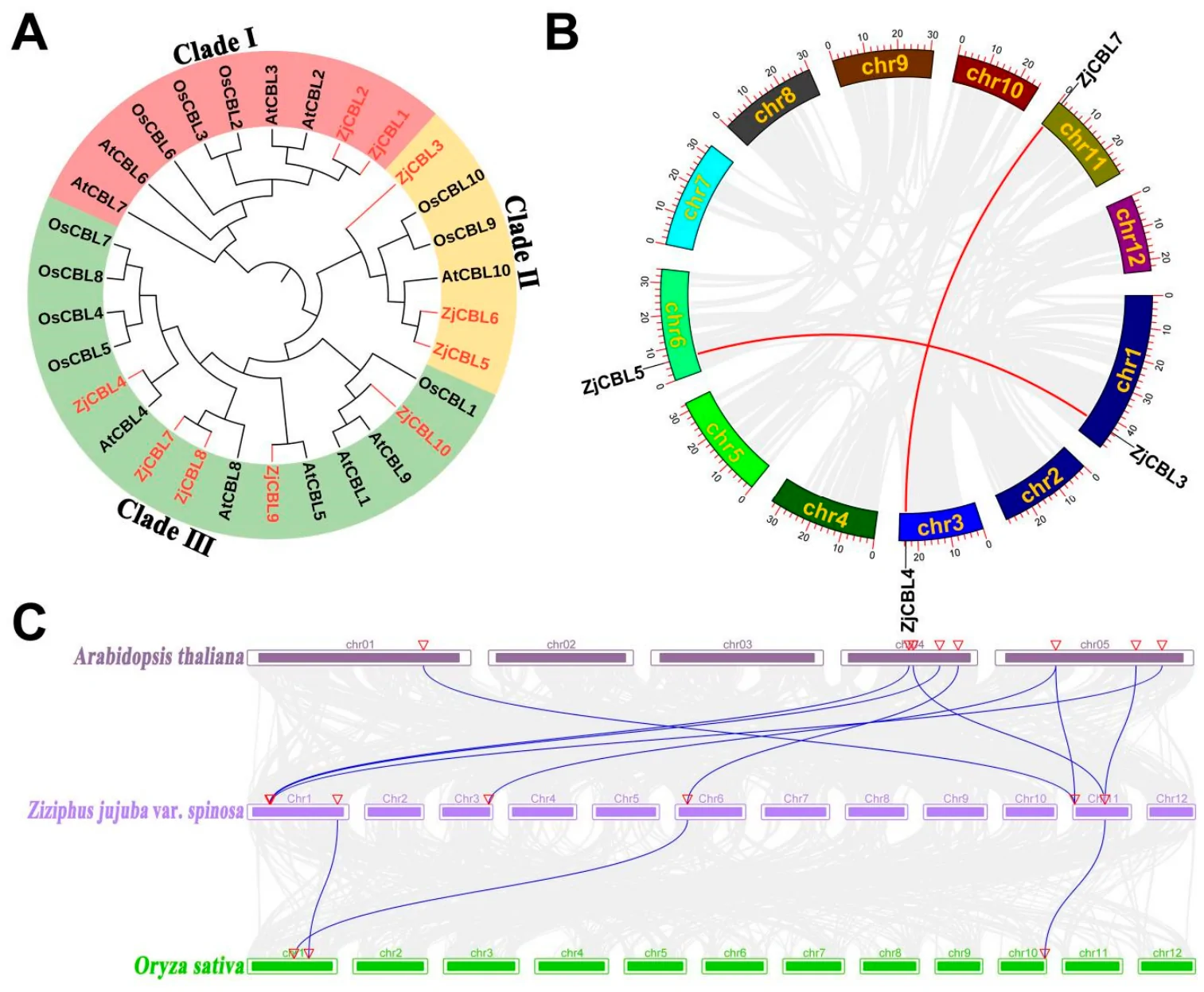
Phylogenetic and collinearity analysis of the ZjCBLs. (**A**) Phylogenetic analysis of CBLs in *Ziziphus jujuba* var. *spinosa*, *Arabidopsis thaliana*, and *Oryza sativa*. (**B**) Intraspecific collinearity analysis of ZjCBLs. Red lines represent ZjCBL gene pairs, and gray lines represent homologous blocks in the background of the sour jujube genome. (**C**) Collinearity analysis of CBLs across three plant species. The blue lines highlight the collinear blocks in the genomes of sour jujube and the other two plant species.

To elucidate the evolutionary relationships of ZjCBL family members, an ML phylogenetic tree was constructed using CBL protein sequences from sour jujube, *A. thaliana*, and *O. sativa* (Figure 1A). The 10 ZjCBL proteins were grouped into three well-supported distinct clades. Clade III contained the most ZjCBL members (ZjCBL4, ZjCBL7, ZjCBL8, ZjCBL9), while Clade I and Clade II each included 3 members (Clade I: ZjCBL1–ZjCBL3; Clade II: ZjCBL5, ZjCBL6, ZjCBL10). Consistent with species evolutionary relationships, ZjCBLs shared higher sequence homology with CBLs from the dicot *A. thaliana* than with those from the monocot *O. sativa*.

Intraspecific collinearity analysis identified two collinear ZjCBL gene pairs in the sour jujube genome: *ZjCBL3*/*ZjCBL5* and *ZjCBL4*/*ZjCBL7* (Figure 1B). To further explore the evolutionary patterns and selection pressure acting on *ZjCBL* genes, we calculated the Ka and Ks substitution rates and Ka/Ks ratios for each collinear pair. All collinear ZjCBL pairs had Ka/Ks ratios far below 1 (Table 2), indicating that these duplicated gene pairs have undergone strong purifying selection during evolution. Interspecific collinearity analysis further revealed that 6 *ZjCBL* genes formed 9 collinear pairs with 8 *AtCBL* genes, while only 3 *ZjCBL* genes formed 3 collinear pairs with 3 *OsCBL* genes. The greater number of collinear *CBL* homolog pairs between sour jujube and *A. thaliana* is consistent with the closer evolutionary relatedness of the two dicot species (Figure 1C).

### 3.3. Conserved Motif and Gene Structure Analyses of ZjCBL Genes

**Figure 2:**
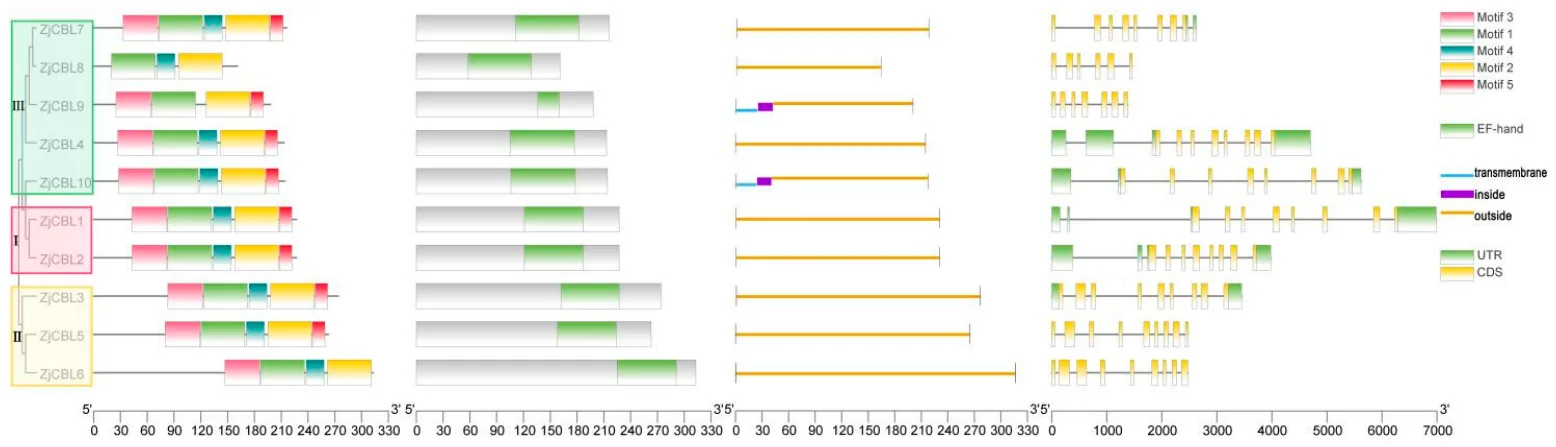
Motif composition, conserved domains, transmembrane topology and gene structure of *ZjCBL* genes.

To further characterize the structural diversity and evolutionary traits of ZjCBL family members, we integrated phylogenetic clustering with analyses of conserved motifs, functional domain architecture, transmembrane topology, and exon–intron organization (Figure 2). Proteins assigned to the same phylogenetic clade exhibited highly consistent motif composition and linear arrangement. All ZjCBL proteins harbored the canonical EF-hand calcium-binding domain, which is a defining feature of the CBL protein family. Transmembrane topology prediction revealed that only two members (ZjCBL4 and ZjCBL9) contain an N-terminal transmembrane helix, whereas all other family members lack transmembrane segments. At the genomic level, ZjCBL genes varied substantially in total length, ranging from approximately 1 kb to over 7 kb, with each gene harboring 6 to 9 exons, and genes assigned to the same phylogenetic clade exhibited highly similar exon–intron distribution patterns.

### 3.4. Promoter Cis-Acting Element Analysis of ZjCBL Genes

**Figure 3:**
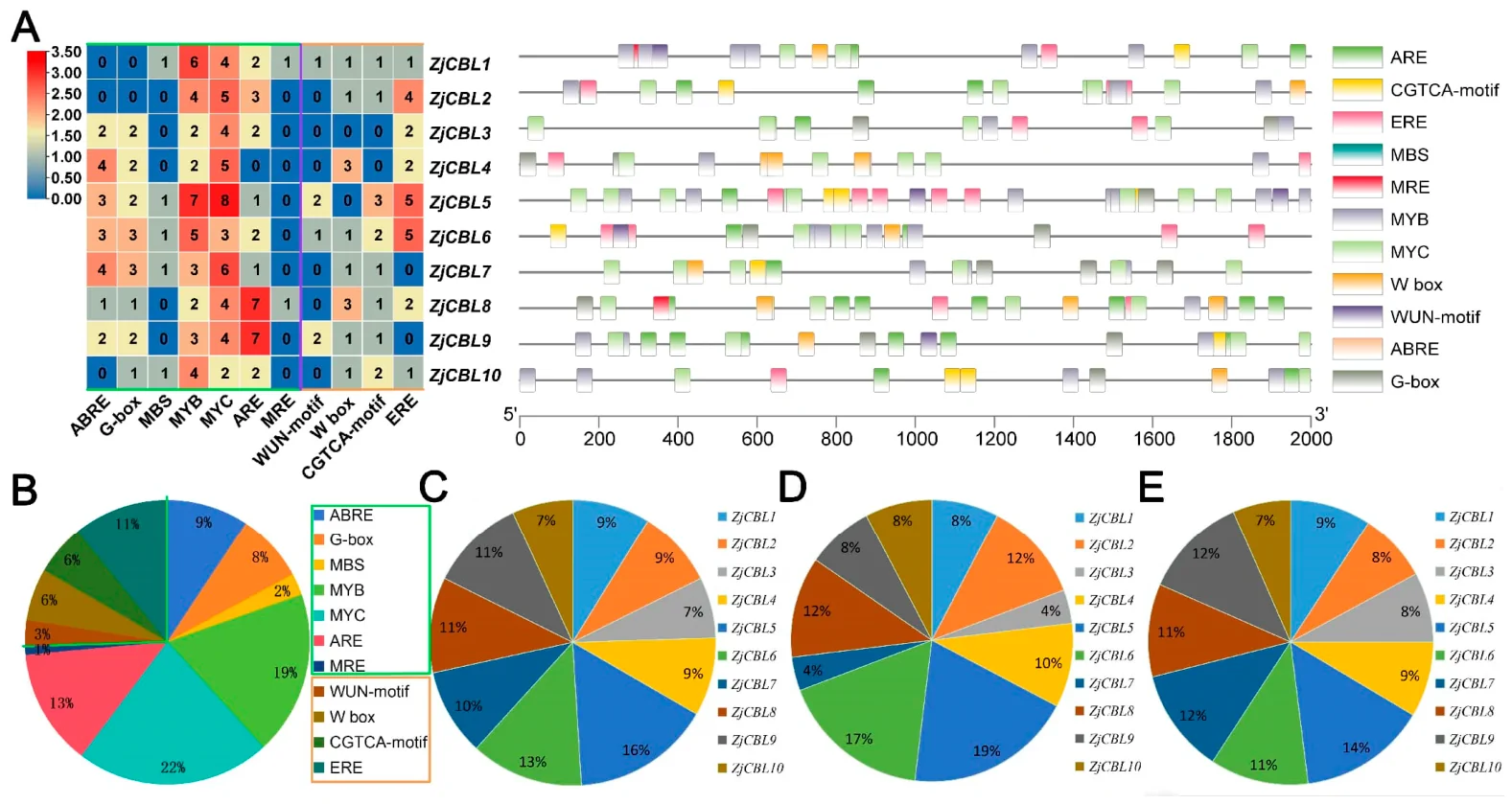
Promoter cis-acting element analysis of *ZjCBLs*. (**A**) Distribution and quantitative statistics of cis-acting elements in *ZjCBL* promoters. (**B**) Relative abundance of distinct cis-acting element types detected across all *ZjCBL* promoters. (**C**–**E**) Pie charts illustrating the percentage distribution of total stress-associated cis-elements (**C**), biotic stress-responsive elements (**D**), and abiotic stress-responsive elements (**E**) for separate *ZjCBL* family members.

To dissect the transcriptional regulatory characteristics of *ZjCBL* genes, we profiled biotic and abiotic stress-responsive cis-acting elements in the 2000 bp promoter regions of all 10 family members (Figure 3). Two major categories of stress-responsive cis-elements were widely distributed across *ZjCBL* promoters: abiotic stress-responsive elements were dominant, accounting for 74.5% of all identified elements, while biotic stress-responsive elements made up the remaining 25.5% (Figure 3A,B). For abiotic stress-related elements, MYC and MYB binding sites, which mediate responses to drought, cold, salinity, and other abiotic stresses, were the most abundant, accounting for 22% and 19% of the total, respectively. Among biotic stress-responsive elements, the ethylene-responsive element (ERE), which participates in pathogen defense pathways, accounted for the highest proportion (11%). The overall repertoire of stress-responsive elements differed significantly among individual *ZjCBL* genes (Figure 3C). *ZjCBL5* (16%) and *ZjCBL6* (13%) contained the highest total number of stress-related elements, and these two genes also had the highest proportion of biotic stress-related elements (19% and 17%, respectively) (Figure 3D). By contrast, abiotic stress-related elements were relatively evenly distributed across all family members, with proportions ranging from 7% to 14% per gene (Figure 3E).

### 3.5. Prediction of miRNA-Target Regulatory Pairs for ZjCBL Genes

To investigate post-transcriptional regulatory mechanisms of *ZjCBL* genes, we predicted miRNA-target interaction pairs and identified three credible regulatory relationships (Table 3). Zju-MIR397a, Zju-MIR160e, and Zju-MIR2950a target *ZjCBL1*, *ZjCBL2*, and *ZjCBL3*, respectively, with expectation scores of 5, 4.5, and 4.5, and each pair has a distinct binding site on the target mRNA. Zju-MIR397a and Zju-MIR160e are predicted to repress their targets via mRNA cleavage, whereas Zju-MIR2950a mediates translational repression of *ZjCBL3*.

### 3.6. Functional Enrichment Analysis of ZjCBL Genes

**Figure 4:**
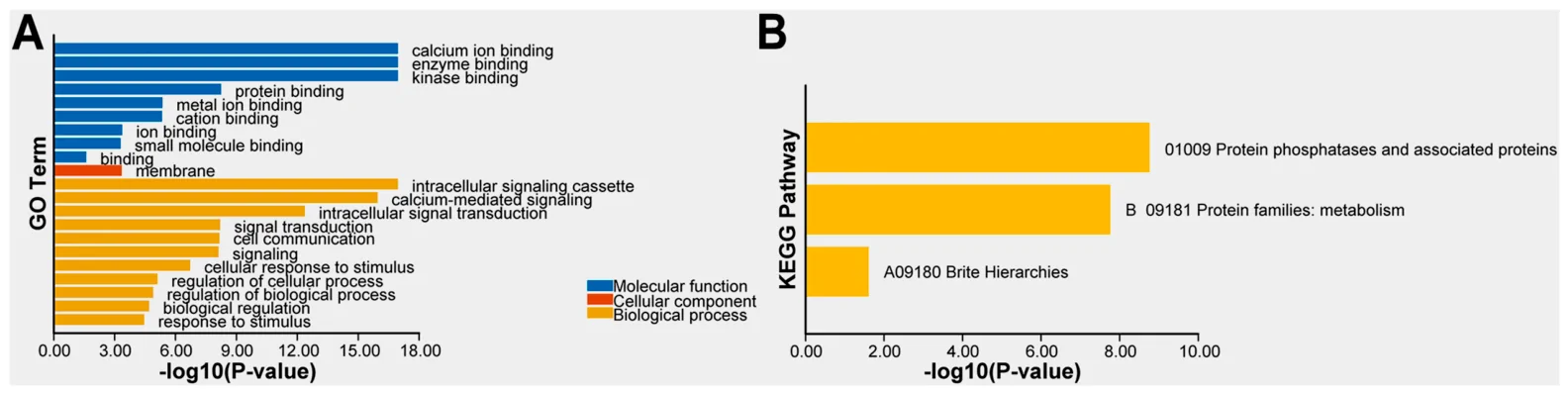
GO and KEGG enrichment analyses of *ZjCBL* gene family. (**A**) Top significantly enriched GO terms grouped into molecular function (blue), cellular component (red), and biological process (yellow); the horizontal axis represents −log_10_(*p*-value). (**B**) Top enriched KEGG pathways, with horizontal axis denoting −log_10_(*p*-value) of enrichment significance.

To explore the biological functions of ZjCBL proteins, we performed GO and KEGG pathway enrichment analyses (Figure 4). In the molecular function category, terms related to calcium ion binding, kinase binding, and protein binding showed the highest enrichment significance. The cellular component category yielded only one significantly enriched term: membrane. For biological processes, terms associated with calcium-mediated signaling, intracellular signal transduction, and stimulus response were the most enriched, which is consistent with the core calcium-sensing function of the CBL family. Subsequent KEGG enrichment identified three significantly enriched pathways, among which “Protein phosphatases and associated proteins” and “Protein families: metabolism” showed the strongest statistical significance. These results indicate that ZjCBL proteins may participate in intracellular signal transduction and metabolic regulation via interactions with phosphatase proteins.

### 3.7. Expression Pattern Analysis of ZjCBL Genes

To explore the functional divergence of the *ZjCBL* family, we analyzed expression profiles and expression correlations across diverse tissues, fruit developmental stages, and multiple abiotic stress and hormone treatments using transcriptomic and qRT-PCR data.

**Figure 5:**
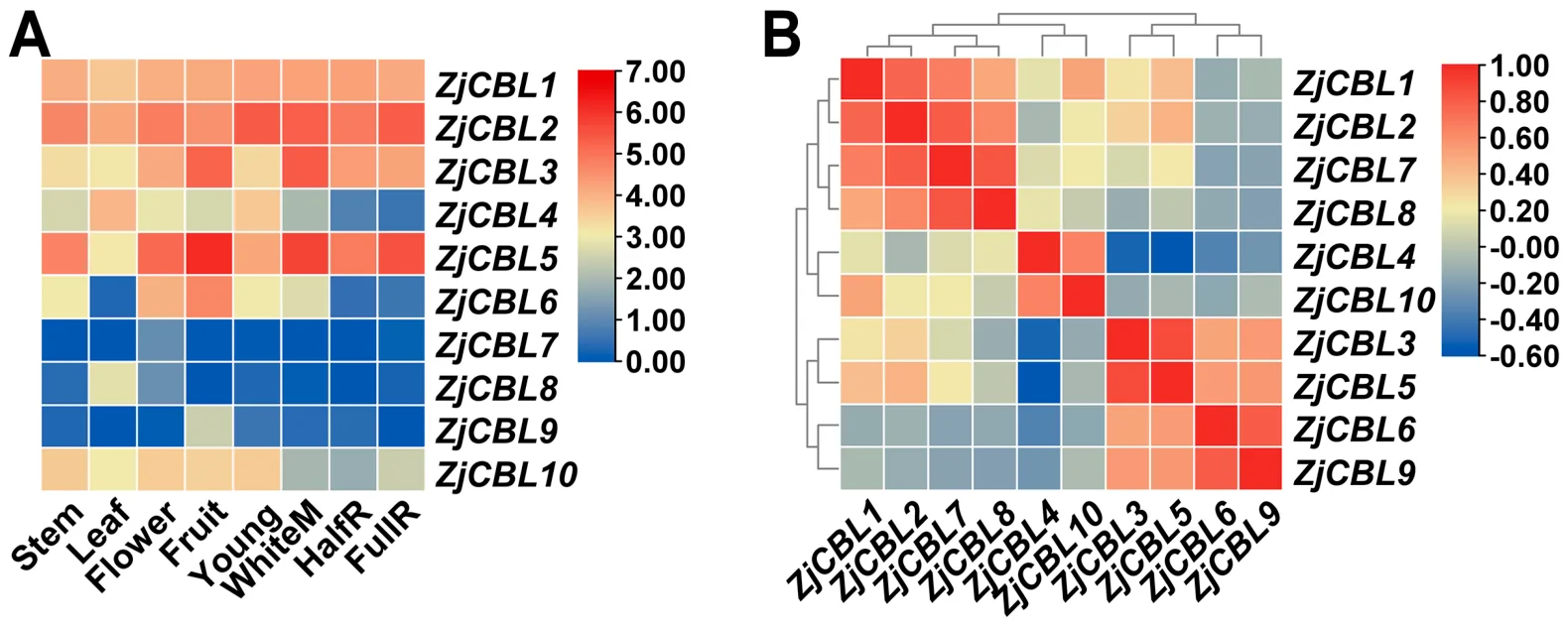
Expression profiles and correlation analysis of *ZjCBL* genes. (**A**) Expression heatmap of *ZjCBLs* in different tissues and fruit developmental stages. (**B**) Pearson correlation coefficient heatmap of *ZjCBLs*, with hierarchical clustering based on expression patterns.

The results revealed that *ZjCBL1*, *ZjCBL2*, *ZjCBL3*, and *ZjCBL5* maintained consistently high transcript levels across different tissues and fruit developmental stages, whereas *ZjCBL7*, *ZjCBL8*, and ZjCBL9 were barely detectable in these samples (Figure 5A). Pearson correlation analysis (N = 35, two-tailed) revealed significant positive expression relationships between several gene pairs: *ZjCBL1* and *ZjCBL2* (*r* = 0.770, *p* < 0.01), *ZjCBL7* and *ZjCBL8* (*r* = 0.832, *p* < 0.01), *ZjCBL3* and *ZjCBL5* (*r* = 0.880, *p* < 0.01), *ZjCBL4* and *ZjCBL10* (*r* = 0.652, *p* < 0.01), as well as *ZjCBL6* and *ZjCBL9 (r* = 0.807, *p* < 0.01) (Figure 5B, Table S4).

**Figure 6:**
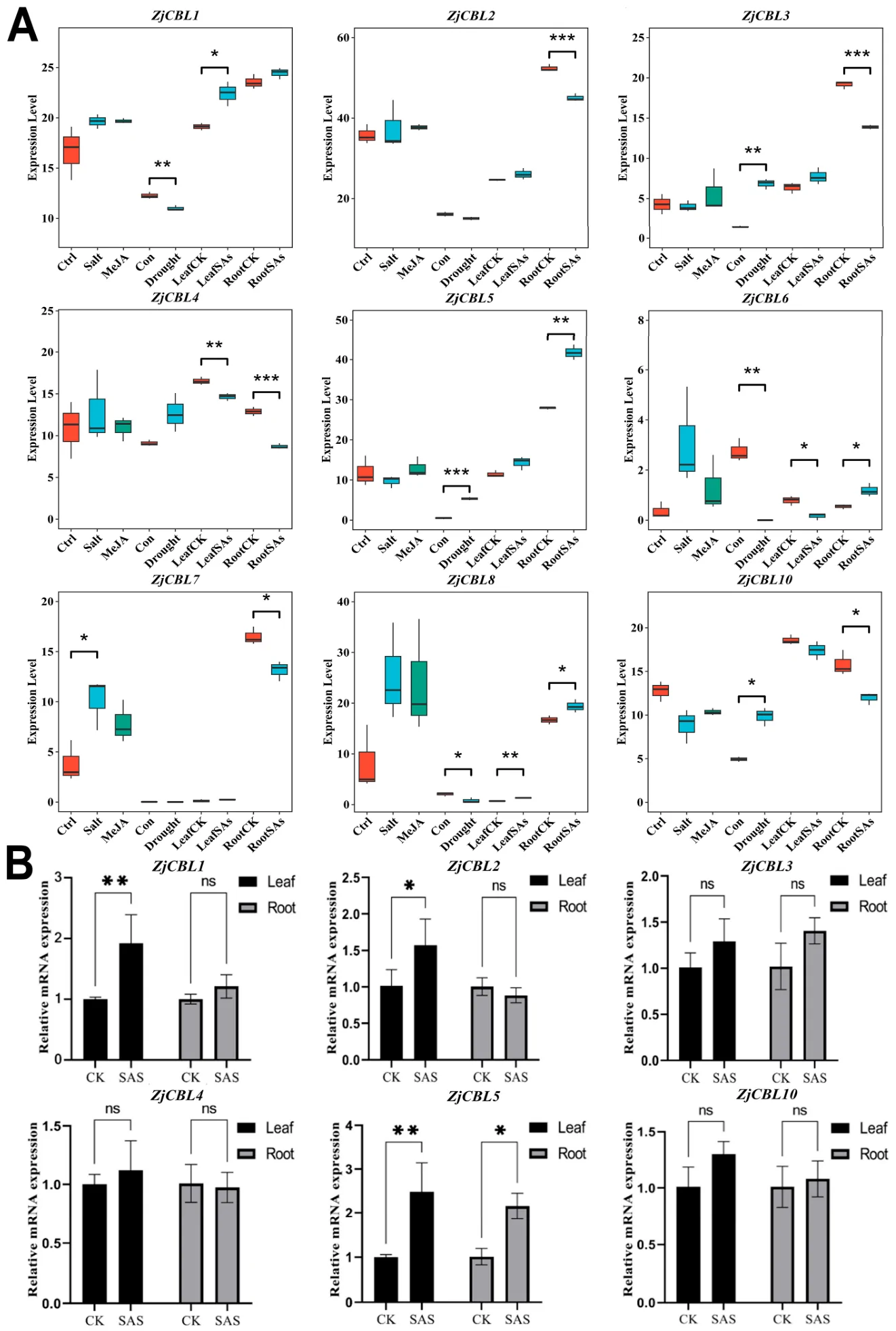
Differential expression analysis of *ZjCBL* genes under various abiotic stress conditions. (**A**) Transcriptional expression patterns of *ZjCBLs* in response to salt, drought, methyl jasmonate (MeJA), and saline–alkali stresses. (**B**) Differential expression analysis of *ZjCBLs* under saline–alkali stress. Asterisks indicate statistically significant differences (* *p* < 0.05; ** *p* < 0.01; *** *p* < 0.001); ns, not significant.

We further assessed *ZjCBL* transcriptional responses to four stress treatments: salt, drought, MeJA, and saline–alkali stress (Figure 6A). Transcriptomic data showed that *ZjCBL1*, *ZjCBL6*, *ZjCBL7*, and *ZjCBL8* were induced under salt and MeJA treatments. Under drought stress, *ZjCBL3*, *ZjCBL5*, and *ZjCBL10* were significantly upregulated, while *ZjCBL1*, *ZjCBL6*, and *ZjCBL8* were strongly downregulated. Saline–alkali stress triggered tissue-specific expression changes: in leaves, *ZjCBL1* and *ZjCBL8* were strongly induced, while *ZjCBL4* and *ZjCBL6* were repressed; in roots, *ZjCBL5*, *ZjCBL6*, and *ZjCBL8* were significantly upregulated, whereas *ZjCBL2*, *ZjCBL3*, *ZjCBL4*, *ZjCBL7*, and *ZjCBL10* were markedly downregulated. For qRT-PCR validation (Figure 6B), we focused on six *ZjCBL* genes that exhibited detectable expression levels in the RNA-seq data under saline–alkali stress; the remaining four genes were excluded from qRT-PCR analysis due to very low or undetectable transcript levels in the tested tissues and difficulties in primer design. In general, the qRT-PCR results confirm the tissue-specific induction trends observed in the RNA-seq data: *ZjCBL1*, *ZjCBL2*, and *ZjCBL5* were significantly upregulated in leaves, and *ZjCBL5* was also strongly induced in roots. However, some quantitative discrepancies were noted between the two platforms, particularly in the magnitude of fold-change, which likely reflects methodological differences in sensitivity and normalization rather than biologically inconsistent patterns. Collectively, these findings indicate that *ZjCBL* family members exhibit condition- and tissue-specific stress responsiveness, suggesting their potential roles in mediating abiotic stress adaptation (Figure 6B).

### 3.8. Dynamic Transcript and Physiological Responses of ZjCBL Genes Under Saline–Alkali Stress

**Figure 7:**
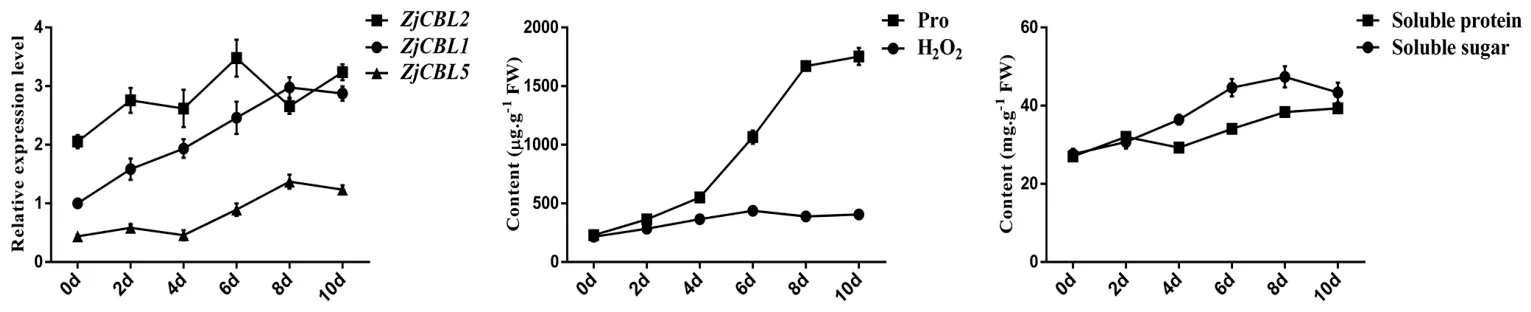
Dynamic changes in *ZjCBL* gene expression and stress-related physiological indicators under continuous stress treatment over 10 days.

To characterize the dynamic expression of *ZjCBL* genes and coupled physiological changes in sour jujube leaves under continuous saline–alkali stress, we performed a 10-day stress treatment to quantify transcript levels of three strongly induced genes (*ZjCBL1*, *ZjCBL2*, *ZjCBL5*), along with four physiological indicators: Pro, H_2_O_2_, soluble protein, and soluble sugar (Figure 7). All three *ZjCBL* genes were significantly upregulated with prolonged stress, but showed distinct expression kinetics: *ZjCBL1* transcript levels increased steadily from day 0 to day 8 and declined slightly at day 10; *ZjCBL2* had a higher overall expression baseline with fluctuating induction, peaking at day 6; *ZjCBL5* showed the mildest induction amplitude, reaching maximum transcript abundance at day 8.

Physiological indicator changes were consistent with *ZjCBLs* transcriptional trends: Pro content increased sharply across the treatment period and peaked at day 10, while H_2_O_2_ levels rose moderately and plateaued after day 6. Soluble protein and soluble sugar concentrations accumulated gradually under stress, indicating that these osmoprotective metabolites accumulate to alleviate stress-induced cellular damage. The temporal association between *ZjCBL* transcript upregulation and the accumulation of osmoprotective metabolites suggests a possible contribution of ZjCBL proteins to plant acclimation to adverse conditions by modulating osmotic balance and maintaining ROS homeostasis, although formal correlation analysis would be required to establish a quantitative relationship.

### 3.9. Yeast Two-Hybrid Verification of ZjCBL1-ZjCIPK Interactions

**Figure 8:**
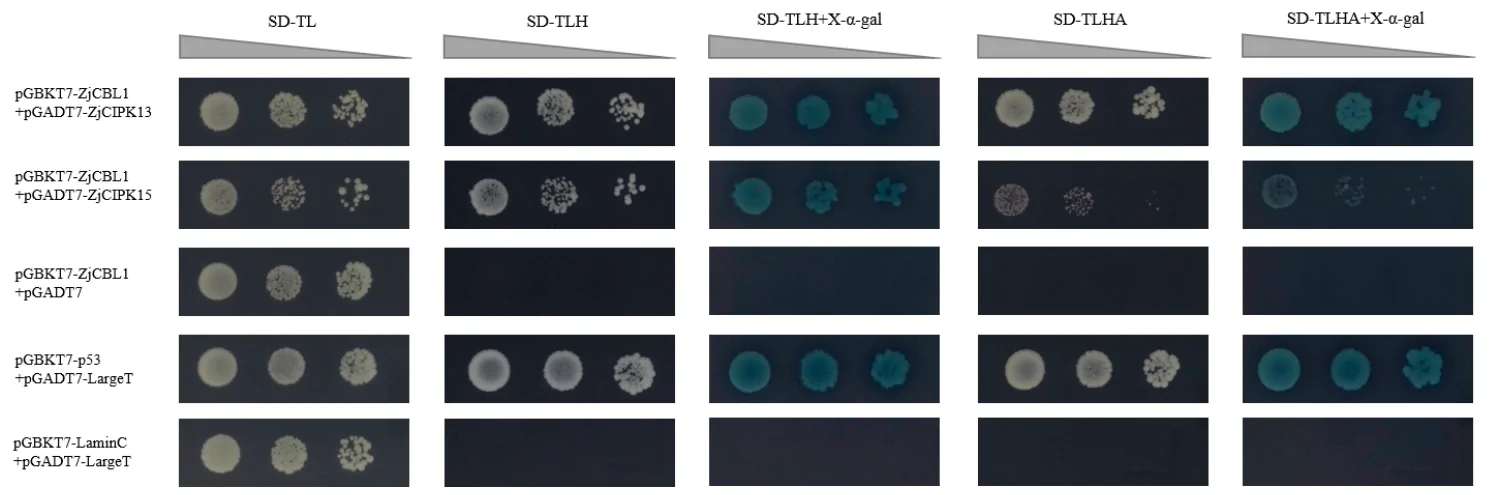
Yeast two-hybrid validation of physical interactions between ZjCBL1 and ZjCIPK proteins in AH109 yeast. Serial 10-fold dilutions (10^0^, 10^−1^, 10^−2^) of AH109 co-transformants were spotted onto SD-TL, SD-TLH, SD-TLH supplemented with X-α-gal, SD-TLHA, and SD-TLHA supplemented with X-α-gal selective media. *pGBKT7-ZjCBL1* co-transformed with empty *pGADT7* served as the autoactivation control; *pGBKT7-p53* + *pGADT7-LargeT* and *pGBKT7-LaminC* + *pGADT7-LargeT* were used as positive and negative interaction controls, respectively. Blue colony staining indicates positive protein–protein physical interaction after 6 days incubation at 30 °C.

CBL proteins exert their functions by forming complexes with CIPKs to mediate abiotic stress responses. To verify physical interactions between ZjCBL1 and two candidate ZjCIPKs in AH109 yeast cells, we performed Y2H assays using ZjCBL1 as bait and ZjCIPK13 and ZjCIPK15 as prey (Figure 8). All AH109 co-transformants grew normally on SD-TL medium, confirming successful co-transformation, as validated by colony PCR. On selective SD-TLH plates, yeast co-expressing ZjCBL1 with either ZjCIPK13 or ZjCIPK15 formed distinct colonies that turned blue on SD-TLH medium supplemented with X-α-gal; identical staining phenotypes were observed on the more stringent SD-TLHA medium containing X-α-gal. The autoactivation control (*pGBKT7-ZjCBL1* + empty *pGADT7*) only produced colonies on SD-TL plates and showed no growth on SD-TLH or SD-TLHA, ruling out intrinsic transcriptional activation of the ZjCBL1 bait construct. Assay validity was supported by standard controls: the positive control (*pGBKT7-p53* + *pGADT7-LargeT*) developed clear blue colonies across all selective media, whereas the negative control (*pGBKT7-LaminC* + *pGADT7-LargeT*) failed to grow on any triple or quadruple dropout plates. Collectively, these AH109 Y2H results confirm physical binding between ZjCBL1 and both ZjCIPK13 and ZjCIPK15. Based on colony density and blue color intensity across serial 10-fold dilutions (10^0^, 10^−1^, 10^−2^), the interaction signal of ZjCBL1 with ZjCIPK13 was slightly stronger than that with ZjCIPK15.

## 4. Discussion

Ca^2+^ ions function as ubiquitous second messengers that govern a wide spectrum of physiological processes in plants, encompassing growth, development, and adaptive responses to environmental stimuli [34]. As a class of Ca^2+^ sensors, CBLs perceive and transduce calcium signals by physically interacting with their downstream target kinases, CIPKs, thereby initiating downstream phosphorylation cascades [12]. Although the CBL gene family has been extensively characterized in model species such as *A. thaliana* and *O. sativa*, revealing its fundamental importance in plant life activities [35,36], no systematic investigation of the CBL family has been reported in sour jujube to date.

In this study, a comprehensive genome-wide survey identified ten non-redundant ZjCBL proteins in sour jujube. Although this number is lower than that reported in herbaceous species such as *Medicago sativa* (23 members) and *Chenopodium quinoa* (16 members) [28,29], it is comparable to the CBL family sizes observed in several woody perennials, including *Citrus sinensis* (8), *Carya illinoinensis* (9), *Camellia sinensis* (8), and *Magnolia biondii* (6) [19,20,21,22]. This discrepancy in family size likely reflects species-specific whole-genome duplication events and lineage-specific gene loss during long-term evolutionary trajectories.

Gene duplication generates paralogous copies that are subject to degenerative mutations; however, purifying selection eliminates deleterious variants and constrains functional divergence, thereby exerting stabilizing effects on gene family evolution [37]. In the present study, the Ka/Ks ratios for all collinear ZjCBL gene pairs were substantially below 1, indicating that the expansion of the ZjCBL family has been predominantly driven by strong purifying selection. This evolutionary pattern is consistent with observations in the CBL families of *Carya illinoinensis* and *Medicago sativa*, which similarly experienced intense purifying selection during their evolutionary histories [20,28]. Interspecific synteny analysis revealed that sour jujube shares a greater number of homologous CBL gene pairs with *A. thaliana* than with *O. sativa*, corroborating its phylogenetic placement within dicotyledons and reflecting the evolutionary conservation of core Ca^2+^ signaling components across dicot lineages. Conserved motif and gene structure analyses further substantiated the evolutionary conservation of the ZjCBL family. All ZjCBL proteins harbored intact canonical EF-hand Ca^2+^-binding motifs, a hallmark structural feature of the CBL family. Members situated within the same phylogenetic clade exhibited similar motif arrangements and exon–intron architectures. Moreover, all ZjCBL proteins were predicted to localize to the plasma membrane, consistent with subcellular localization profiles reported for CBL proteins in most other plant species [38,39]. Notably, ZjCBL4 and ZjCBL9 contain N-terminal transmembrane helices, which may mediate specialized membrane-anchoring modes and confer distinct signal transduction capacities.

Cis-acting elements within promoter regions regulate gene expression and thereby participate in various biological processes, including plant growth, development, and stress responses [39,40,41]. Phytohormones play pivotal roles in regulating plant growth, development, and stress adaptation [42]. Previous studies have established that CBL proteins participate in diverse biological processes via the ABA signaling pathway; for instance, overexpression of *ZmCBL9* enhances plant tolerance to ABA stress [27]. In the present study, the promoters of ZjCBL genes were found to be enriched in hormone- and stress-responsive regulatory sequences, including MYB/MYC binding sites, ABRE, and ERE elements. The widespread distribution of these cis-elements provides a mechanistic explanation for the robust transcriptional induction of multiple *ZjCBL* genes under saline–alkali, drought, and MeJA treatments, and further suggests that ZjCBL-mediated Ca^2+^ signaling is intimately linked with ABA and jasmonic acid (JA) hormonal networks under stress conditions [43].

Transcriptomic and qRT-PCR analyses revealed that *ZjCBL* genes exhibit distinct tissue- and stress-specific expression patterns. *ZjCBL1*, *ZjCBL2*, *ZjCBL3*, and *ZjCBL5* maintained consistently high transcript levels across various tissues and throughout fruit developmental stages, implying that these genes participate in basal Ca^2+^ signaling essential for normal growth and development. By contrast, *ZjCBL7*–*ZjCBL9* were expressed at extremely low levels in all tissues examined, suggesting that they may be specifically activated only upon external stress perception [44,45]. Correlation analysis of gene expression identified multiple significantly positively correlated gene pairs, such as *ZjCBL1*/*ZjCBL2* and *ZjCBL7*/*ZjCBL8*, suggesting the possibility of shared upstream regulatory mechanisms. However, correlation analysis alone cannot confirm transcriptional co-regulation or functional redundancy; further experimental evidence, including promoter binding assays and reverse genetic approaches, is required to test these inferences.

Under saline–alkali stress, *ZjCBL* genes exhibited pronounced tissue-specific expression changes. In leaves, *ZjCBL1* and *ZjCBL8* were rapidly and strongly upregulated, whereas root-specific induction was observed predominantly for *ZjCBL5*, *ZjCBL6*, and *ZjCBL8*. This tissue-specific activation pattern implies that ZjCBL-mediated Ca^2+^ signaling may fulfill distinct physiological functions in above-ground versus below-ground organs. In leaves, *ZjCBL* family members likely participate in mitigating oxidative damage induced by saline–alkali stress, whereas in roots, they may primarily function in sensing extracellular ionic imbalance and transducing early stress signals [46]. Environmental stresses typically provoke alterations in plant phenotypes and physiological and biochemical characteristics [47,48]. Time-series expression analysis under sustained saline–alkali treatment further identified *ZjCBL1* as a core stress-responsive gene, exhibiting sustained upregulation throughout the initial 8-day treatment period with a substantial magnitude of induction. The expression dynamics of *ZjCBL1* were observed to coincide temporally with the accumulation patterns of proline and soluble sugars in leaves. This temporal association hints at a possible role for ZjCBL1 in osmotic adjustment, but we acknowledge that formal correlation analysis has not been performed, and further experiments—such as targeted metabolite profiling in transgenic lines—are needed to establish a causal relationship between *ZjCBL1* expression and osmoprotectant accumulation.

CBL proteins must form functional signaling complexes with CIPK protein kinases to transduce intracellular Ca^2+^ signals, a mode of action that has been extensively validated [49,50]. In sour jujube, expression data indicated that *ZjCBL1* exhibited significant induction under saline–alkali stress, positioning it as a candidate component of the ZjCBL–ZjCIPK regulatory network. Y2H assays confirmed that ZjCBL1 physically interacts with both ZjCIPK13 and ZjCIPK15. Previous studies have demonstrated that AtCBL1/AtCBL9 interact with AtCIPK23 to regulate K^+^ and NO_3_^−^ homeostasis [35], and also associate with AtCIPK26 to phosphorylate RBOHF, thereby modulating ROS signaling pathways [51]. It is plausible that upon Ca^2+^ binding via its EF-hand motifs, ZjCBL1 undergoes conformational changes that relieve the autoinhibitory domain of ZjCIPK13 and ZjCIPK15. The activated kinase complexes would then be expected to phosphorylate downstream ion transporters and stress-responsive transcription factors, coordinately regulating intracellular ion homeostasis and antioxidant defense systems. However, these proposed phosphorylation events and downstream targets were not directly examined in this study and remain to be validated in future work. Semiquantitative assessment of Y2H colony growth on gradient dilution plates further revealed differential interaction strengths between the two complexes: ZjCBL1 exhibited a slightly stronger interaction signal with ZjCIPK13 than with ZjCIPK15, suggesting functional bias in the Ca^2+^ signaling pathways mediated by these two complexes in sour jujube. As a naturally saline–alkali-tolerant woody germplasm, the ZjCBL1–ZjCIPK13/15 interaction modules identified in this study provide critical molecular insights into the mechanistic basis of its robust stress tolerance.

In summary, this study presents a comprehensive genome-wide characterization of the ZjCBL family, encompassing phylogenetic relationships, gene duplication patterns, cis-regulatory features, transcriptional regulation, tissue- and stress-dependent expression dynamics, and core protein–protein interactions. The stress-inducible genes *ZjCBL1*, *ZjCBL2*, and *ZjCBL5* identified herein represent promising candidate targets for molecular breeding aimed at enhancing stress tolerance in horticultural crops. Nevertheless, several limitations warrant acknowledgment. First, the Y2H assays only validated two ZjCIPK interactors for ZjCBL1; comprehensive interactome mapping, such as yeast two-hybrid library screening or bimolecular fluorescence complementation (BiFC) assays, will be required to delineate the complete ZjCBL–ZjCIPK interaction network in sour jujube. Second, all functional predictions are currently based on bioinformatic analyses and heterologous Y2H validation; stable overexpression or gene-silencing transgenic lines in sour jujube are needed to corroborate the in vivo biological functions of target *ZjCBL* genes under saline–alkali stress. Third, the downstream substrates phosphorylated by ZjCIPK13 and ZjCIPK15 remain unidentified; future phosphoproteomic approaches may elucidate their downstream signaling targets. Collectively, this study establishes a solid foundation for deciphering Ca^2+^ signaling regulatory networks and developing stress-resilient genetic resources in sour jujube.

## 5. Conclusions

This study presents the first genome-wide identification of the *CBL* gene family in sour jujube, with ten non-redundant *ZjCBL* genes identified and unevenly distributed across four chromosomes, and the annotation error of *ZjCBL1* corrected through molecular cloning. The *ZjCBL* gene family has evolved under strong purifying selection, and all ZjCBL proteins contain highly conserved EF-hand Ca^2+^-binding motifs, while their promoter regions are enriched with diverse stress- and hormone-responsive cis-acting elements. Transcriptomic and qRT-PCR analyses identified divergent expression profiles among *ZjCBL* family members. *ZjCBL1*, *ZjCBL2*, *ZjCBL3*, and *ZjCBL5* showed constitutively high expression levels in various tissues, suggesting their involvement in basal Ca^2+^ signaling essential for normal growth and development. Notably, *ZjCBL1* was identified as a core responsive gene under prolonged saline–alkali stress, with its expression dynamics temporally associated with the accumulation of osmoprotectants in leaves. Yeast two-hybrid assays confirmed that ZjCBL1 physically interacts with two ZjCIPK proteins, ZjCIPK13 and ZjCIPK15, with a stronger interaction signal for ZjCIPK13. Collectively, these findings elucidate the evolutionary features and stress-responsive patterns of the *ZjCBL* gene family in sour jujube, establish *ZjCBL1* as a promising candidate for molecular breeding aimed at enhancing stress tolerance in jujube, and provide a solid foundation for further dissection of the ZjCBL–ZjCIPK regulatory network underlying saline–alkali tolerance in woody plants.

## Figures and Tables

**Table 1:** Physicochemical properties of *ZjCBL* gene family members.

Gene Name	C.L.	Length (aa)	MW (kDa)	pI	S.L.	GeneID ^2^	Identity ^3^
*ZjCBL1*	Chr01	226	25.84	4.75	CM, Cyt ^1^	ZspiChr1G00009600.1	45.87%
*ZjCBL2*	Chr01	226	26.05	4.91	CM	ZspiChr1G00009780.1	100.00%
*ZjCBL3*	Chr01	273	31.68	5.26	CM	ZspiChr1G00023970.1	100.00%
*ZjCBL4*	Chr03	212	24.42	4.82	CM	ZspiChr3G00243980.1	100.00%
*ZjCBL5*	Chr06	262	30.58	4.71	CM, Cyt	ZspiChr6G00036370.1	100.00%
*ZjCBL6*	Chr06	312	36.45	5.18	CM, Cyt	ZspiChr6G00036380.1	100.00%
*ZjCBL7*	Chr11	215	24.96	4.83	CM, Cyt	ZspiChr11G00189760.1	100.00%
*ZjCBL8*	Chr11	160	18.51	5.05	CM, Cyt	ZspiChr11G00189770.1	100.00%
*ZjCBL9*	Chr11	197	22.31	4.7	CM, Cyt	ZspiChr11G00189780.1	100.00%
*ZjCBL10*	Chr11	213	24.49	4.71	CM	ZspiChr11G00197330.1	100.00%

^1^ CM, cell membrane; Cyt, cytoplasm. ^2^ Gene ID, identity code of *ZjCBLs* in the latest released version of a sour jujube genome [32]. ^3^ Identity, sequence identity between the corrected *ZjCBL* CDS sequences and the original *ZjCBL* coding regions in the sour jujube genome [32].

**Table 2:** Evolutionary selection pressure information of *ZjCBLs*.

Gene Pairs	Ka	Ks	Ka/Ks
*ZjCBL3/ZjCBL5*	0.2499	1.6119	0.1551
*ZjCBL4/ZjCBL7*	0.2154	1.0896	0.1977

**Table 3:** Prediction of miRNA target sites in *ZjCBL* genes.

miRNA_Acc.	Target_Acc.	Expectation	Target Site (bp)	Inhibition
*Zju-MIR397a*	*ZjCBL1*	5	171–191	Cleavage
*Zju-MIR160e*	*ZjCBL2*	4.5	9–29	Cleavage
*Zju-MIR2950a*	*ZjCBL3*	4.5	244–264	Translation

## Data Availability

The transcriptome data generated in this study have been deposited in NCBI SRA under BioProject accession PRJNA1440647, PRJNA1078886, PRJNA748347, PRJNA306374, and PRJNA542987.

## Supplementary Materials

The following supporting information can be downloaded at: https://www.mdpi.com/article/10.3390/genes17091121/s1: Table S1: Formula of different saline–alkali solutions; Table S2: Genome annotation information of the *ZjCBL* gene family in sour jujube; Table S3: Detailed information of CBL in phylogenetic tree; Table S4: Pearson correlation coefficients of expression levels among *ZjCBL* genes; Table S5: Primers used for qRT-PCR, gene cloning and vector construction in this study.

## Abbreviations

The following abbreviations are used in this manuscript:

qRT-PCR	Quantitative real-time polymerase chain reaction
ROS	Reactive oxygen species
H_2_O_2_	Hydrogen peroxide
X-gal	5-bromo-4-chloro-3-indolyl-β-D-galactopyranoside
